# New Evidence on the Biobehavioral Mechanisms of Chronic Pain in Aging African Americans

**DOI:** 10.1093/geroni/igaa057.2839

**Published:** 2020-12-16

**Authors:** Staja Booker

## Abstract

African American older adults are living longer with chronic pain, which presents a huge personal and societal burden. A growing group of scholars are now devoted to accurately and precisely characterizing and phenotyping the experience of pain in aging using within-group and advanced methodological designs to elucidate the biopsychosocial-behavioral responses to pain. In this symposium, five dynamic presenters present new evidence on mechanisms of pain in older African-Americans. Dr. Roach’s investigation reveals the effect of genetic alterations of sickle cell disease (SCD) on stress-related pain in younger and older adults; this scientific inquiry is especially important because there is little research on SCD in aging. Next, Dr. Terry, extends these findings by exploring the association between psychosocial factors such as experiences of discrimination, pain catastrophizing, and perceived stress on neural (brain) responses via magnetic resonance imaging. From a clinical perspective, Dr. Booker reports on the first-ever model of intra-racial differences in movement-evoked pain in older African-Americans with knee osteoarthritis and healthy controls. Our final two presenters use a translational approach to identify how older African-Americans cope with chronic pain. Dr. Robinson-Lane’s study highlights the unique experience and predictors of coping, adaptation, and self-management of chronic pain in Black dementia caregivers. Finally, Dr. Cobb’s research from a large cross-sectional study correlates social, behavioral, and health factors with opioid and psychotropic use in economically disadvantaged older African-Americans. This symposium offers novel ways of understanding social determinants of pain and assisting African-Americans and their caregivers to manage complex chronic pain in later life.

